# Visualization of Film Formation Process of Copolyesteramide Containing Phthalazine Moieties During Interfacial Polymerization

**DOI:** 10.3390/membranes15080233

**Published:** 2025-08-01

**Authors:** Zeyuan Liu, Hailong Li, Qian Liu, Zhaoqi Wang, Danhui Wang, Peiqi Xu, Xigao Jian, Shouhai Zhang

**Affiliations:** 1 State Key Laboratory of Fine Chemicals, School of Chemical Engineering, Dalian University of Technology, Dalian 116024, China; liuzeyuan@mail.dlut.edu.cn (Z.L.); lihailong@dlut.edu.cn (H.L.); liuqian-chem@dlut.edu.cn (Q.L.); wangzhaoqi@fudan.edu.cn (Z.W.); wangdh@dlut.edu.cn (D.W.); 18342272606@163.com (P.X.); jian4616@dlut.edu.cn (X.J.); 2 Dalian Key Laboratory of Membrane Materials and Membrane Processes, Liaoning Province Technology Innovation Center of High Performance Resin Materials, Dalian 116024, China

**Keywords:** copolyesteramide, optical three-dimensional digital microscope, interfacial polymerization, co-solvent, film morphology

## Abstract

Interfacial polymerization (IP) has been widely utilized to synthesize composite membranes. However, precise control of this reaction remains a challenge due to the complexity of the IP process. Herein, an optical three-dimensional microscope was used to directly observe the IP process. To construct copolyesteramide containing phthalazine moiety films, rigid monomer 4-(4′-hydroxyphenyl)-2,3-phthalazin-1-one (DHPZ) and flexible monomer piperazine (PIP) were used as aqueous phase monomers, and trimesoyl chloride (TMC) served as the organic phase monomer. Multilayer cellular structures were observed for the copolyesteramide films during the IP process. The effects of multiple factors including the ratio between flexible and rigid monomers, co-solvents, and the addition of phase transfer catalysts on the film growth and the morphologies were investigated. This research aims to deepen our understanding of the IP process, especially for the principles which govern polymer film growth and morphology, to promote new methodologies for regulating interfacial polymerization in composite membrane preparation.

## 1. Introduction

Membrane separation technology has received considerable attention in the industrial fields since the late 1960s. Its energy efficiency and environmentally friendly properties make it a viable replacement for conventional methods such as distillation, evaporation, adsorption, and extraction [1,2,3]. However, significant improvements are still needed in various membrane technologies in terms of separation performance, energy demand, and cost [4]. State-of-the-art reverse osmosis (RO) and nanofiltration (NF) membranes generally adopt a thin-film composite (TFC) architecture [5,6]. This structure incorporates a polyamide layer (10–300 nm thick) formed directly on top of a porous substrate via interfacial polymerization (IP) [3,6,7]. These membranes achieve high salt rejection, high water permeability, and operational stability under high-pressure conditions. Generally speaking, the interfacial polymerization process is carried out by two highly reactive monomers at the interface of two immiscible solutions. The interfacial polymerization reaction is fast and has self-limiting properties [5,8]. There are many reaction parameters that can be adjusted, such as monomer structure and concentration, polymerization time, additives, etc. Different reaction conditions interact with each other, making it difficult to optimize membrane performance. Therefore, it is of great importance to extensively investigate the effects of these factors on the interfacial polymerization process.

Researchers have conducted numerous experiments to investigate the IP process. Previous research methods such as light reflection (LRI), pendant drop tension (PDR), and pendant drop mechanical analysis (PDMA) often measured various properties (e.g., optical properties, interfacial tension, stress relaxation of suspended drops, etc.) of polymer films indirectly, making it difficult to intuitively study the formation process of polymer films and record their fine structures [9,10,11,12]. Observing the growth of IP films using an optical microscope provides us with the possibility of understanding the formation process of IP films [13,14]. Inspired by those works, a new methodology to follow the IP process was recently built in our research group using an optical three-dimensional digital microscope (OTM). Xu et al. [15] used an OTM to observe the interfacial polymerization of 2,4-(4-hydroxyphenyl)-2,3-phthalazin-1-one (BPPZ) with TMC, or IPC, or TPC. The influence of phase transfer catalysts or organic phase solvents on the film formation process and film morphology was studied. An OTM is a type of microscopic imaging technology that can scan and record the morphology of thin films, thereby better studying the interface polymerization reaction process.

The role of co-solvents in interfacial polymerization is widely recognized due to their ability to expand the reaction zone by reducing the solubility disparity and interfacial tension between aqueous and organic phases [16,17,18]. This enlarged reaction zone promotes the formation of a more pronounced ridge-and-valley topography compared to conventional structures, resulting in a less dense crosslinked polymer network and a multilayered, porous polyamide (PA) layer [16,17,19,20,21,22]. As a consequence, the permeability of the membranes typically increases. However, it is also feasible that the selective layer could become thicker as a result of the excessive growth of the membrane structures, consequently diminishing water transport efficiency. Given the complexity involved in studying the formation patterns, it remains crucial to explore the mechanisms underlying these morphological transformations.

The aim of this study is to visualize the process of interface aggregation to form copolyesteramide containing phthalazine moiety films, which were generated through interfacial polymerization reaction using rigid twisted monomer 4-(4′-hydroxyphenyl)-2,3-phthalazin-1-one (DHPZ) and flexible monomer piperazine (PIP) as aqueous monomers, and triphenyl chloride (TMC) as an organic monomer. An OTM was used to follow the IP process in situ. The effects of polymerization time, the monomer ratio, the co-solvent, and the phase transfer catalyst (PTC) on the surface morphologies of copolyester amide films were extensively studied.

## 2. Materials and Methods

### 2.1. Materials

The 4-(4′-hydroxyphenyl)-2,3-phthalazin-1-one (DHPZ) compound was supplied via Dalian Baoli New Material Company (Dalian, China), and Trimesoyl chloride (TMC, purity > 99%) and 4-dimethylaminopyridine (DMAP, purity > 99%) were purchased from TCI Chemical Industry Development Co., Ltd. (Shanghai, China). Piperazine and 18-crown-6 (purity > 99%) were purchased from Aladdin Reagent Co., Ltd. (Shanghai, China). Cyclohexane (CYH, AR) and potassium hydroxide (KOH, AR) were purchased from Tianjin Kemiou Chemical Co., Ltd. (Tianjin, China).

### 2.2. Formation and Characterization of IP Film

**Figure 1:**
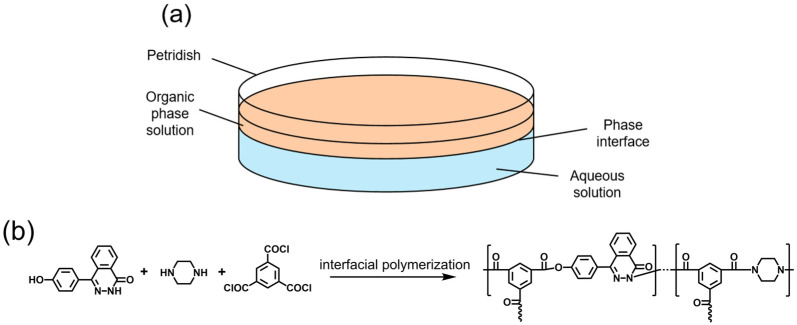
(**a**) Schematic diagram of interfacial polymerization phase interface. (**b**) Interfacial polymerization for preparation of films based on DHPZ, PIP, and TMC.

The experiments were conducted at a relative humidity of 40–50% and a temperature of 25 °C. As shown in Figure 1, a 10.0 mL aqueous solution was first prepared in a 60 mm glass Petri dish. The aqueous monomers were DHPZ and PIP with a total concentration of 0.0019 mol·L^−1^, and the KOH amount was twice the molar ratio of DHPZ. An organic solution of TMC (5 mL, 0.01 mol·L^−1^) was poured onto the surface of the aqueous solution. Chemical functional groups of the films were examined by Fourier-transform infrared spectroscopy (FTIR, Thermo Nicolet IS50, Waltham, MA, USA); the chemical bonding was characterized using X-ray photoelectron spectroscopy (XPS, Thermo Fisher Scientific ESCALAB250Xi, East Grinstead, UK).

### 2.3. Observation of IP Film Morphology

An optical three-dimensional digital microscope (Hirox RH-2000, Tokyo, Japan) was used to record the formation process of the copolyesteramide film during the IP reaction. The reaction time was defined as starting from the moment of contact between the two solutions. Images of 20~50 different focal planes were sent to the OTM’s post-processing software to record every surface morphology. The obtained images of the film were continuously transmitted to the software of the OTM to obtain a three-dimensional model of the membrane, and the surface-area-to-projected-area ratio was calculated by the software. The manual selection tool in the image analysis software ImageJ 1.53e was utilized to outline each discernible irregular elliptical target contour individually, and their area values were measured and recorded. Subsequently, the equivalent circular diameter of each target was calculated based on the area. The equivalent circular diameter values of all measured targets were then summarized, and the mean and standard deviation were calculated as the final statistics.

## 3. Results and Discussion

### 3.1. The Formation Process of Polymer Films

**Figure 2:**
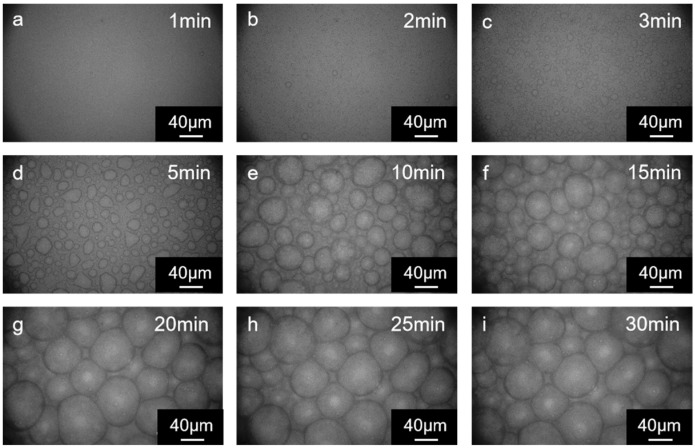
Evolution of cellular structure over reaction time of (**a**) 1 min, (**b**) 2 min, (**c**) 3 min, (**d**) 5 min, (**e**) 10 min, (**f**) 15 min, (**g**) 20 min, (**h**) 25 min, and (**i**) 30 min. (*n* (DHPZ)/*n* (PIP) = 8/2; phase transfer catalyst: 18-crown-6 (0.005 wt%); organic solvent: cyclohexane containing 3 wt% butanone).

**Figure 3:**
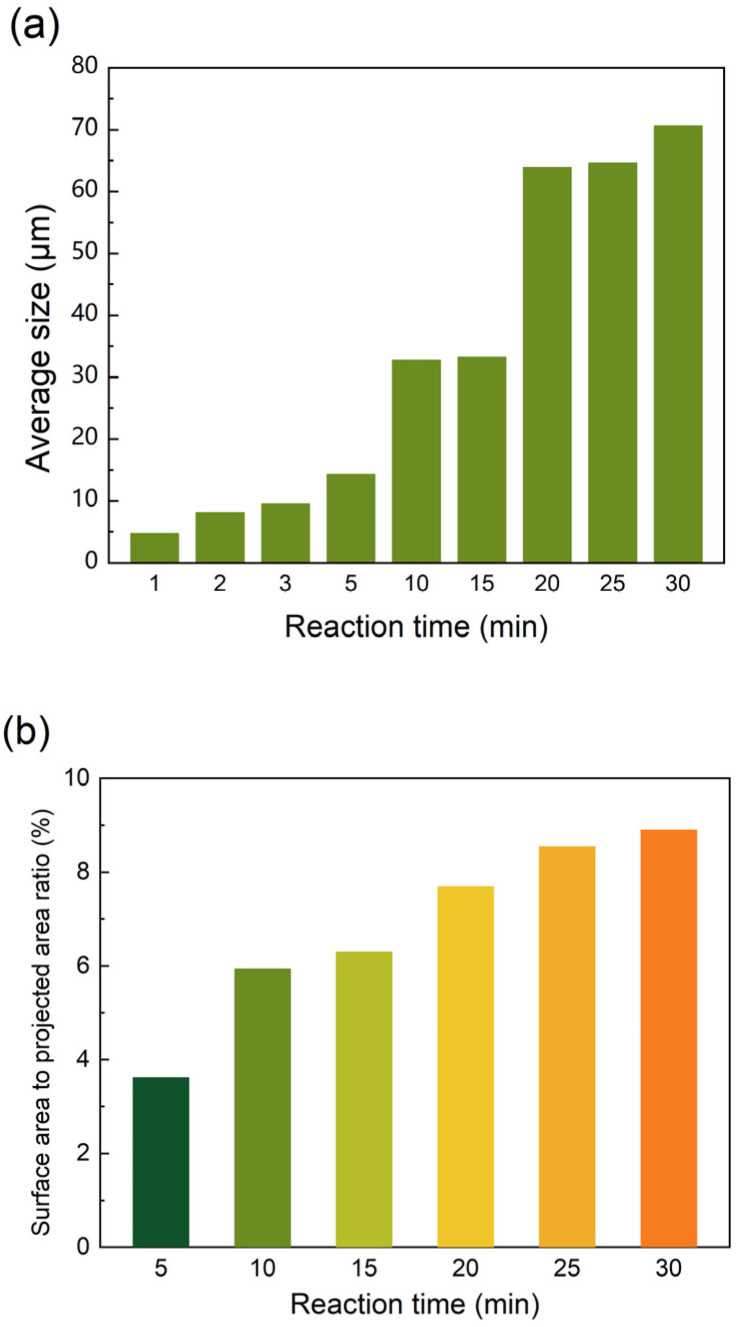
(**a**) The average size of “cellular” structures; (**b**) the surface-area-to-projected-area ratio of the films with reaction times.

The chemical structure of the film was confirmed by FT-IR spectroscopy and XPS analysis (Figures S1 and S2), and was consistent with the expected structure. The reaction process was observed over a period of 30 min and the representative images are shown in Figure 2. In the early stage of the reaction (1–5 min, Figure 2a–d), “cellular” structures were visible within the field of view. As the time progressed from 10 to 20 min (Figure 2e,f), a new layer of larger-sized “cellular” structures formed atop the original cellular structures. By the 20 min mark, a third layer of even larger “cellular” structures emerged, after which the growth of these structures on the film surface largely ceased through to the 30 min mark (Figure 2g–i). To clearly track the changes, the diameters of the “cellular” structures were quantified using ImageJ software (Figure 3a), revealing an increasing trend corresponding to different growth stages of the structures. Based on three-dimensional models, the surface-area to-projected-area ratio of the polymer films was calculated by the software of the OTM (Figure 3b). The results indicated that as the size of the “cellular” structures increased, so did the surface-area-to-projected-area ratio. This increment was particularly significant at the onset of each new growth phase. Given that minimal changes occurred in the film’s growth after 20 min, subsequent investigations will focus solely on the reaction conditions within the first 15 min.

**Figure 4:**
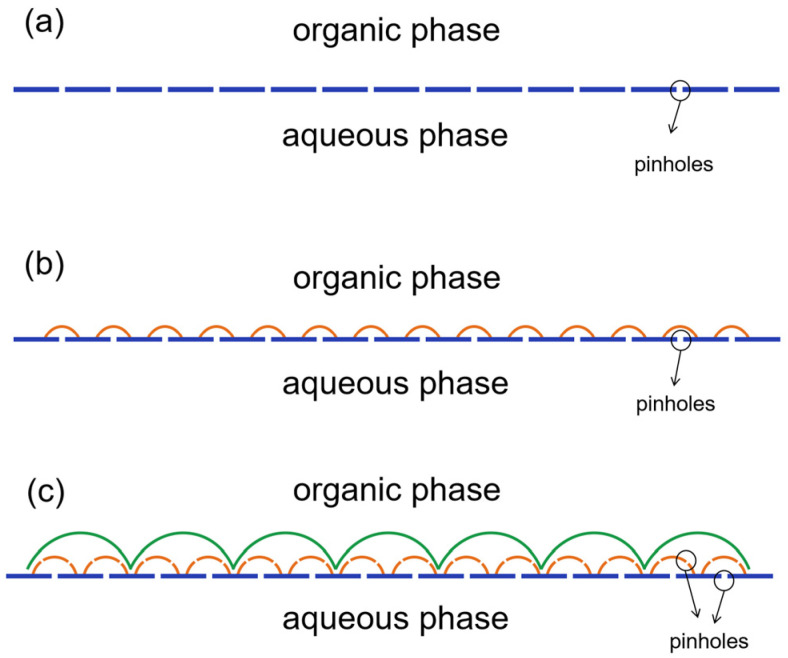
Mechanism of interface polymerization film formation: (**a**) incipient film pinhole structure; (**b**) cellular structures of the first layer; (**c**) cellular structures of the second layer.

The formation of a multilayered “cellular” polymer film morphology may occur as follows: when an aqueous solution comes into contacting with an organic solution, interfacial polymerization begins immediately, leading to the formation of a solid film at the liquid–liquid interface [14]. Due to their large surface-area-to-volume ratio, such films have a large surface energy per unit of volume and are thermodynamically unstable [23]. At this stage, due to the relatively low degree of cross-linking in the polymer [24], the film spontaneously develops numerous uniformly distributed pinhole structures as a means of minimizing surface energy (Figure 4a) [25,26,27,28]. Such films with pinholes can be called incipient films. Because the incipient film is hydrophilic, aqueous solution can then enter the other side of the incipient film, i.e., the organic phase, through these pinhole structures by infiltration [14]. As the aqueous solution penetrates into the organic phase in the form of droplets, it continues to contribute to the growth of the polymer film until it is well established. Consequently, the “cellular” film’s size increases continuously during this period (Figure 4b). This cavity structure with pores at the bottom facilitates an increase in contact area, i.e., an increase in membrane flux, when applied to the membrane separation of liquids. Some studies have shown that the selective layers of the composite membranes exhibit similar structures [22,29].

When the polymer film of the first aqueous droplets across the incipient film is essentially complete, the size of the “cellular” structures will no longer increase. Similarly to the incipient film, these “cellular”-structured films are likewise susceptible to the development of pinholes, which facilitates the formation of subsequent generations of “cellular” structures (Figure 4c). The second layer of “cellular”-structured films is largely independent from the first layer [14]. By adjusting the microscope’s focus, the underlying first layer of “cellular”-structured films remains visible beneath the second layer. The increased size of the second layer of “cellular” structures results from the continuous consumption of aqueous monomers as the aqueous solution penetrates into the organic phase. As the diffusion distance of aqueous-phase monomers into the aqueous/organic interface increases, the supply rate of these monomers decreases, leading to the formation of larger “cellular” structures. Similarly, a third layer of “cellular” structures begins to form at approximately 20 min. However, due to the increased difficulty in supplying aqueous monomers, the “cellular” structures in this layer are larger due to slower film formation. In the third layer, the “cellular” structures begin to squeeze and deform into each other without exhibiting agglomeration. This observation indicates that the “cellular” structures represent a polymer film rather than droplets, although this polymer film forms at a slower rate. Subsequent observations reveal how parameters such as the ratio of rigid/flexible monomers, the co-solvent, and the phase transfer catalyst impact the film formation process.

### 3.2. Aqueous Phase Monomer Ratio

**Figure 5:**
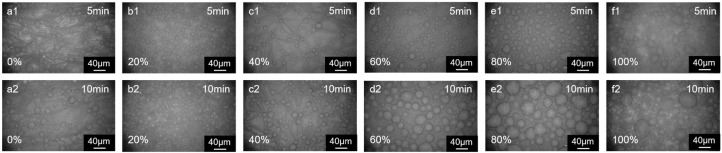
The effect of DHPZ content in the aqueous phase on the morphology of polymer membranes at (**a1**,**a2**) 0%, (**b1**,**b2**) 20%, (**c1**,**c2**) 40%, (**d1**,**d2**) 60%, (**e1**,**e2**) 80%, and (**f1**,**f2**) 100%. Phase transfer catalyst: 18-crown ether-6 (0.005 wt%); organic solvent: cyclohexane containing 3 wt% butanone.

**Figure 6:**
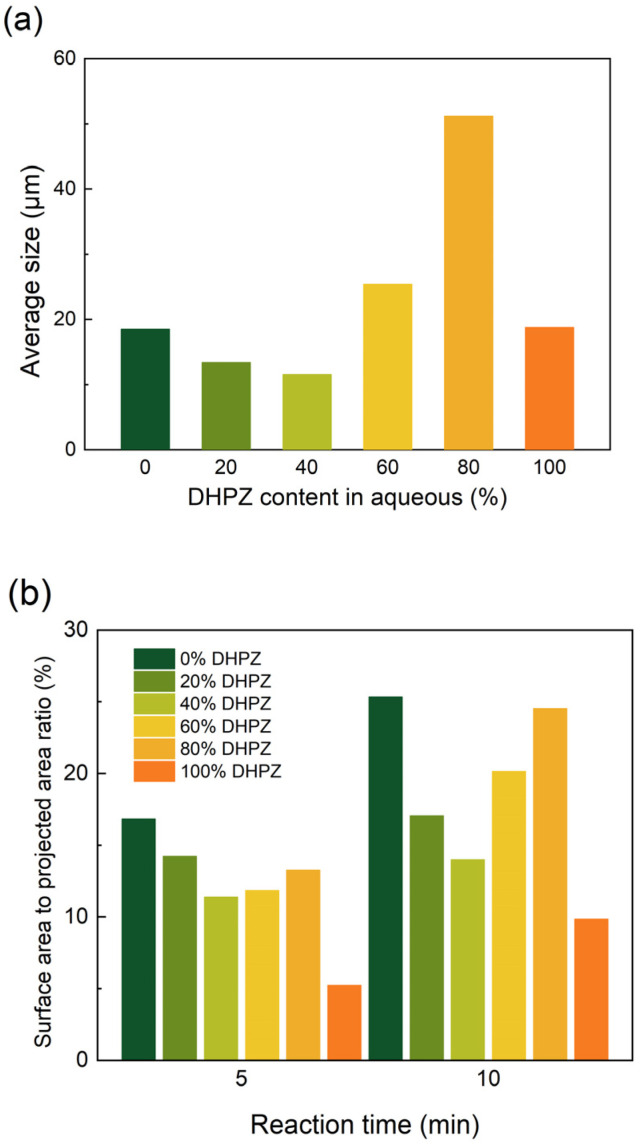
(**a**) The average size of “cellular” structures (10 min); (**b**) the increase in the surface area of the films with the DHPZ ratio in aqueous monomers.

The interfacial polymerization of polymer films with different monomer proportions is shown in Figure 5. When the aqueous phase monomers were all flexible monomers (100% PIP), the resulting polymer films were relatively soft and wrinkles were formed in the incipient film due to the nature of the interfacial instability [30]. Similar wrinkles were observed when the aqueous phase monomers were 80% and 60% PIP (Figure 5(b1,c1,b2,c2)). As the proportion of DHPZ in the aqueous monomer increased (Figure 5(d1,d2,e1,e2)), the incipient film became progressively flatter, and more “cellular” structures appeared (Figure 6a). This is attributed to the slower reaction rate of DHPZ compared to PIP, leading to a slower formation process of the polymer film. When all monomers in the aqueous phase were DHPZ (Figure 5(f1,f2)), the molecular chains of the resulting polymer film exhibited rigid structures, making the film more brittle. This brittleness led to the continuous formation and subsequent collapse of the foam structure. At the boundaries where collapse occurs, the aqueous solution can recontact the organic phase, facilitating the production of smaller “cellular” structures. Unlike other cases, an additional inhomogeneous brightness is observed on the film’s surface. This phenomenon results from the superposition of the collapsed “cellular”-structured films over the primary film.

**Figure 7:**
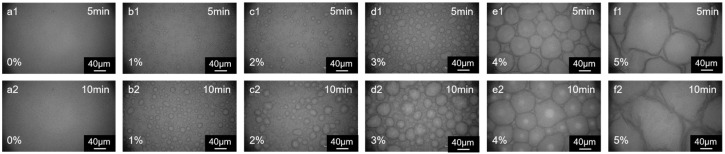
The effect of butanone concentration in the organic phase on the morphology of polymer membranes at (**a1**,**a2**) 0%, (**b1**,**b2**) 1%, (**c1**,**c2**) 2%, (**d1**,**d2**) 3%, (**e1**,**e2**) 4%, and (**f1**,**f2**) 5%. The concentration of monomers in the aqueous phase is 0.0062 mol/L, with *n* (DHPZ)/*n* (PIP) = 8/2; the TMC concentration is 0.0098 mol/L. The KOH concentration of aqueous acid absorbent is 0.001 mol/L; the phase transfer catalyst 18-crown ether-6 concentration is 0.005 wt%.

The surface-area-to-projected-area ratios of the polymer films, as depicted in Figure 7b, are analyzed in relation to the DHPZ ratio. With an increase in the DHPZ ratio, the polymer film tends to exhibit a flatter morphology due to the gradual disappearance of wrinkle structures. Therefore, as the wrinkle structures disappear, the surface area of the polymer film decreases as the DHPZ ratio increases from 0% to 40%. Upon increasing the DHPZ content to 60% or 80%, the growth rate of surface area accelerates over time due to the expansion of the “cellular” films during the reaction. Eventually, this increase in surface area diminishes markedly as a result of the collapse of the “cellular” films. In composite membrane preparation, membranes prepared with the addition of rigid monomers can exhibit higher fluxes compared to those prepared with pure piperazine [31,32]. The variation in the roughness of the membrane surface with the monomer ratio can also show a similar trend to the increase in the surface area of the films in this study [32]. In conclusion, the formation of polymer films through interfacial polymerization is not governed by a single mechanism that is applicable to all monomer types. Monomer properties have a fundamental impact on the formation process of the selective layer of the composite film and are ultimately reflected in the structure and morphology. By adjusting the type and proportion of monomers, properties such as the surface morphology of the composite membrane can be finely controlled simultaneously.

### 3.3. Co-Solvent

**Figure 8:**
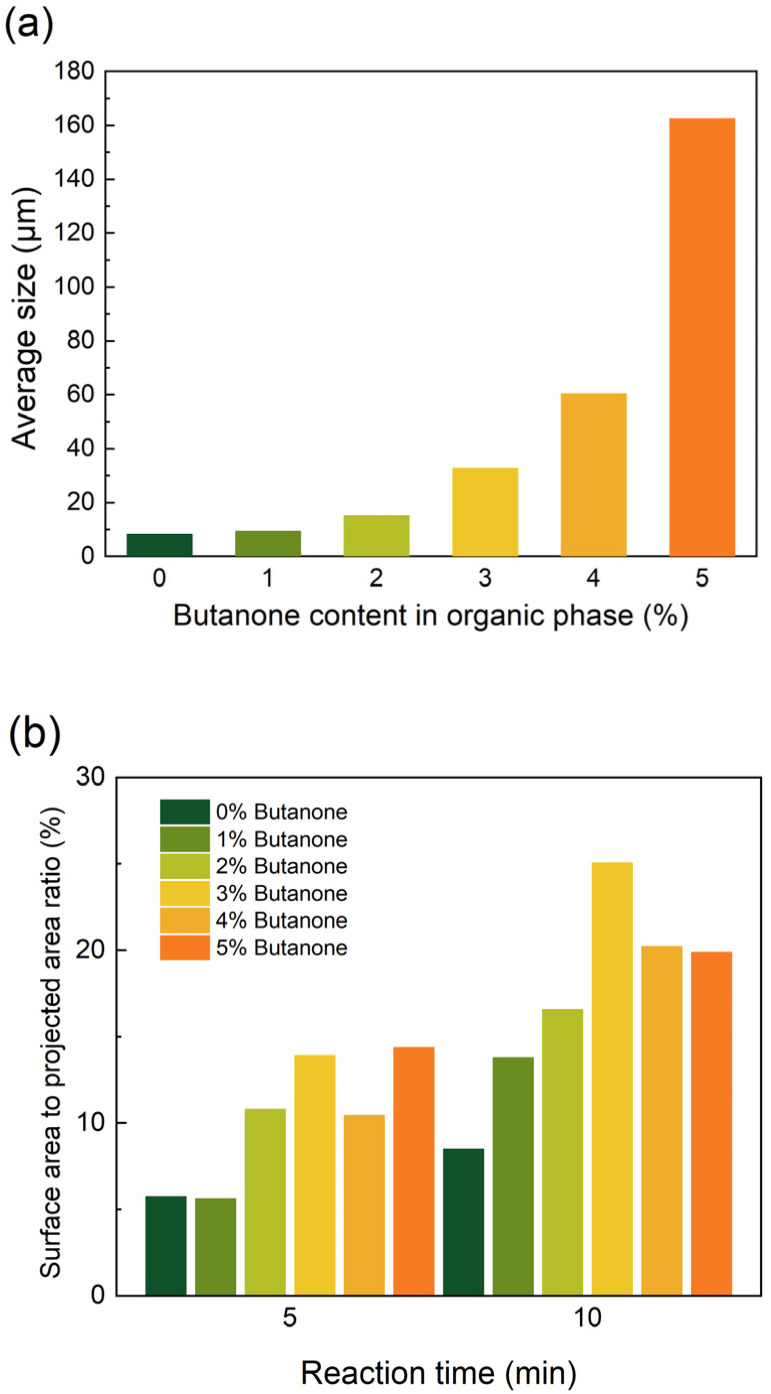
(**a**) The average size of “cellular” structures (10 min); (**b**) the surface-area-to-projected-area ratio of the films versus the butanone content in the organic phase.

As illustrated in (Figure 7(a1,a2)), in the absence of butanone in the organic phase, only a limited number of “cellular” structures were observed on the polymer surface. With an increase in butanone concentration, the size of the “cellular” structures increases. Upon further addition of butanone, reaching concentrations of 4% and 5% (Figure 7(e1,e2,f1,f2)), the size of the “cellular” structures grows significantly (Figure 8a). The addition of a co-solvent is often thought to increase the region of miscibility between the two phases, which usually results in a more relaxed polymer film being formed [33]. This may make it easier for the aqueous phase solution to enter the organic phase through pinholes, with larger droplets of the aqueous phase solution entering the organic phase, and therefore a significant increase in the size of the cell structure. The film surface exhibits a smoother morphology, as indicated by the reduction in surface-area-to-projected-area ratio (Figure 8b), resulting from the reduced gaps within the densely arranged “cellular” structures. This phenomenon cannot be solely attributed to a decreased rate of “cellular” structures. Instead, it is primarily caused by the increased interfacial instability resulting from the diffusion of the co-solvent from the organic phase to the aqueous phase [21]. Nanofiltration (NF) membranes prepared by the co-solvent method using DHPZ and PIP showed a significant increase in flux of 260% compared to membranes prepared without the use of co-solvents, while maintaining excellent salt retention. Na_2_SO_4_ retention was only slightly decreased by 1.3% [34]. The surface morphology of the NF membranes also showed some similarities with the morphology of the membranes used in the present study. This instability leads to the enlargement of pinhole defects, thereby significantly increasing the rate at which droplets transfer from the aqueous phase into the organic phase. In the case of co-solvent-assisted preparation of composite membranes, membranes prepared using pure piperazine show less variation in surface roughness [19]. In contrast, membranes using rigid monomers such as resorcinol can exhibit a more complex surface morphology, and the membrane flux is usually increased [35,36]. Similarly to the monomer property, co-solvents also play a complex role in the preparation of composite membranes. Therefore, when using co-solvent-assisted interfacial polymerization to prepare composite membranes, it is also important to take into account the changes in the morphology of the selective layer, rather than only investigating the correlation between the type of co-solvent and the performance of the composite membrane.

### 3.4. Phase Transfer Catalyst

**Figure 9:**
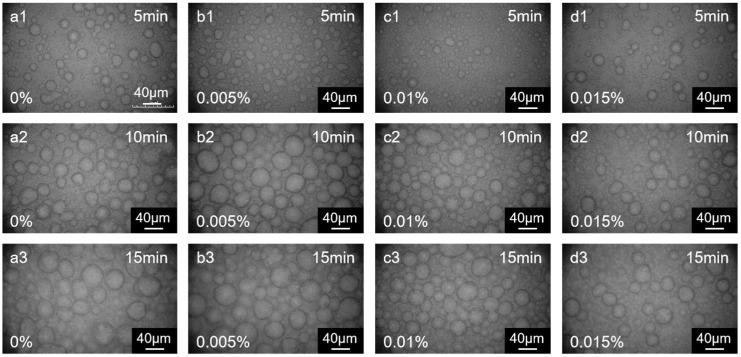
The effect of the concentration of 18-crown ether-6 in the aqueous phase on the morphology of polymer membranes at (**a1**–**a3**) 0 wt%, (**b1**–**b3**) 0.005 wt%, (**c1**–**c3**) 0.010 wt%, and (**d1**–**d3**) 0.015 wt%. *n* (DHPZ)/*n* (PIP) = 8/2; organic solvent: cyclohexane containing 3 wt% butanone.

**Figure 10:**
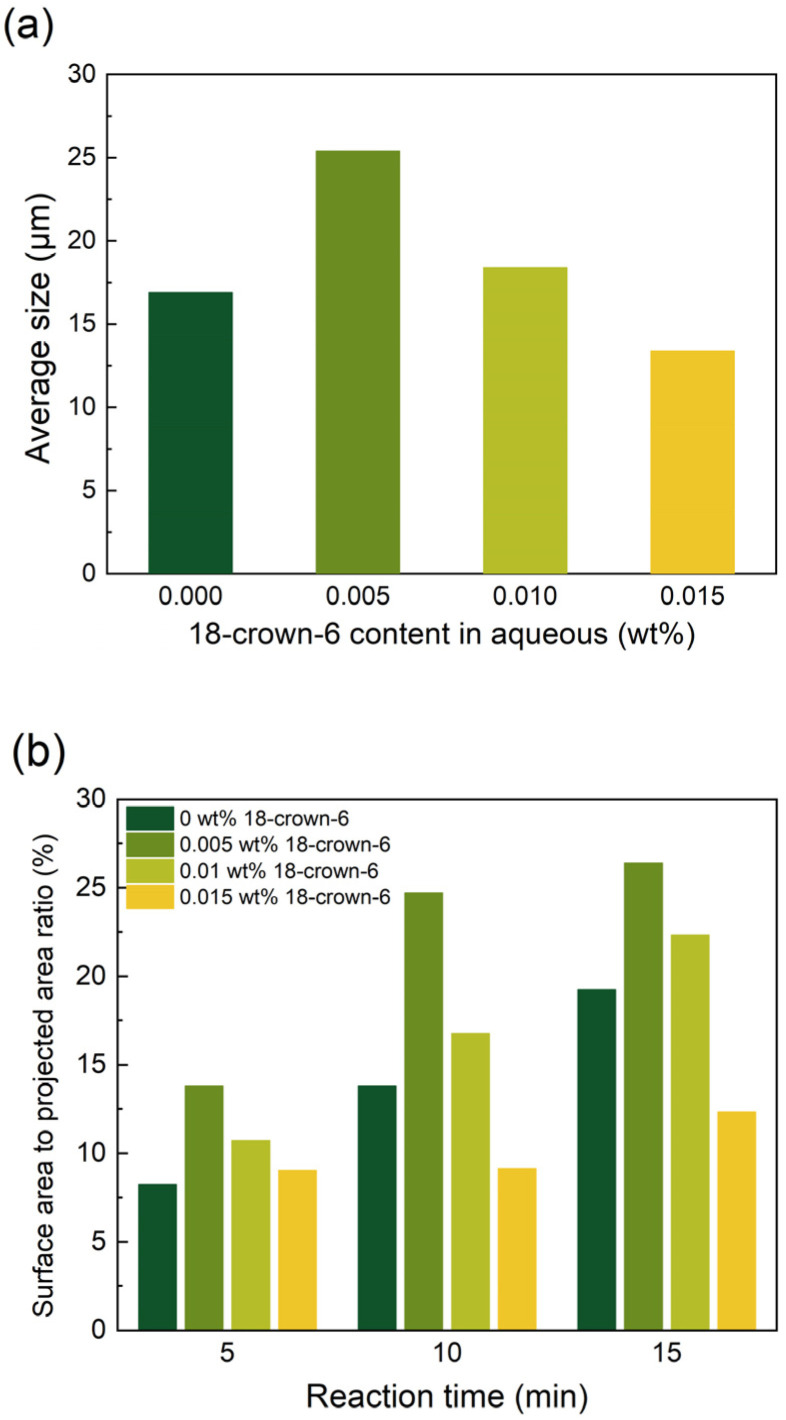
(**a**) The average size of “cellular” structures (10 min); (**b**) the surface-area-to-projected-area ratio of the films versus 18-crown-6 content in the aqueous phase.

The phase transfer catalyst also influences the morphology of the resulting polymer films. The catalyst 18-crown-6 serves as an effective phase transfer catalyst in interfacial polymerization processes [37]. Specifically, as illustrated in Figure 9(a1–a3), the development of “cellular” structures was less pronounced in the absence of 18-crown-6 in the aqueous phase compared to when 0.005 wt% 18-crown-6 was incorporated (Figure 9(b1–b3)). Since the reaction ability of DHPZ was enhanced to a lesser extent, the size of “cellular” structures and surface-area-to-projected-area ratio were slightly lower (Figure 10). Attributed to the relatively increased proportion of PIP participating in the reaction, the “cellular” structures refined at an accelerated rate. As the concentration increased to 0.01 wt% and 0.015 wt% (Figure 9(c1–c3,d1–d3)), the size of the “cellular” films decreased significantly. Since 18-crown-6 increases the reactivity of DHPZ, the rate of refinement of the “cellular” structures also increases. However, this also leads to an earlier appearance of the next generation of “cellular” films. On the membrane surface, the first layer of “cellular” films was relatively smaller in size (Figure 10a), whereas the second generation of “cellular” structures appeared to be more dispersed. Phase transfer catalysts are usually used to enhance the reactivity of interfacial reactions in order to increase the degree of cross-linking of selective layer polymers. However, the interaction between the parameters involved in interfacial polymerization should be considered to improve the overall separation performance of the membrane.

## 4. Conclusions

In this study, the process of DHPZ/PIP-TMC interfacial polymerization was investigated using an optical three-dimensional digital microscope. The results demonstrated that the evolution of film morphology adhered to the mechanism of pinhole formation, cellular growth, and multilayer construction. The pinhole structure in the primary film, induced by surface energy, facilitated the permeation of the aqueous phase. This process subsequently led to the formation of rough, multilayered structures in the secondary film. Modulation of the aqueous monomer ratio revealed that a higher PIP content induced interfacial destabilization and promoted fold formation. In contrast, the increase in the DHPZ ratio suppressed folds but exacerbated pinhole defects, which in turn promoted the regeneration of the cellular structure. The co-solvent facilitates the formation of “cellular” structures by enhancing interfacial perturbation and accelerating monomer mass transfer. However, an excessive amount of co-solvent induces structural over-aggregation and reverses the flattening effect. The phase transfer catalyst 18-crown-6 facilitates DHPZ diffusion and enhances its reactivity with TMC. When the concentration of 18-crown-6 was raised to 0.005 wt%, the growth of “cellular” structures of the primary membrane was promoted. Further increasing the concentration to 0.015 wt% accelerated the growth of these structures, reduced their size, and flattened the overall film morphology. This study confirms that the formation of interfacial polymerization films is a complex process. Enhancing the performance necessitates the synergistic optimization of experimental parameters.

## Data Availability

Data will be made available on request.

## Supplementary Materials

The following supporting information can be downloaded at: https://www.mdpi.com/article/10.3390/membranes15080233/s1, Figure S1: FT-IR spectra of the fabricated films; Figure S2: C 1s XPS spectra of fabricated films (a) n(DHPZ)/n(PIP)=0:10; (b) n(DHPZ)/n(PIP)=8:2; (c) n(DHPZ)/n(PIP)=10:0.
