# Supplementary material for: Visualization of Film Formation Process of Copolyesteramide Containing Phthalazine Moieties During Interfacial Polymerization
Source: Membranes (Basel). 2025 Aug 1;15(8):233. doi: 10.3390/membranes15080233 (PMC12388542; doi:10.3390/membranes15080233)
Supplement: Supplementary file 1 [file membranes-15-00233-s001.zip › membranes-3738699-supplementary.pdf]

Visualization of Film Formation Process of Copolyesteramide Containing Phthalazine Moieties During Interfacial Polymerization

Zeyuan Liu [1,2], Hailong Li [1,2], Qian Liu [1,2], Zhaoqi Wang [1,2], Danhui Wang [1,2], Peiqi Xu [1,2], Xigao Jian [1,2] and Shouhai Zhang [1,2,*]

[1] State Key Laboratory of Fine Chemicals, School of Chemical Engineering, Dalian University of Technology, Dalian 116024, China; liuzeyuan@mail.dlut.edu.cn (Z.L.); lihailong@dlut.edu.cn (H.L.); liuqian-chem@dlut.edu.cn (Q.L.); wangzhaoqi@fudan.edu.cn (Z.W.); wangdh@dlut.edu.cn (D.W.); 18342272606@163.com (P.X.); jian4616@dlut.edu.cn (X.J.)

[2] Dalian Key Laboratory of Membrane Materials and Membrane Processes, Liaoning Province Technology Innovation Center of High Performance Resin Materials, Dalian 116024, China

* Correspondence: zhangshh@dlut.edu.cn; Tel.: +86-411-8498-6107

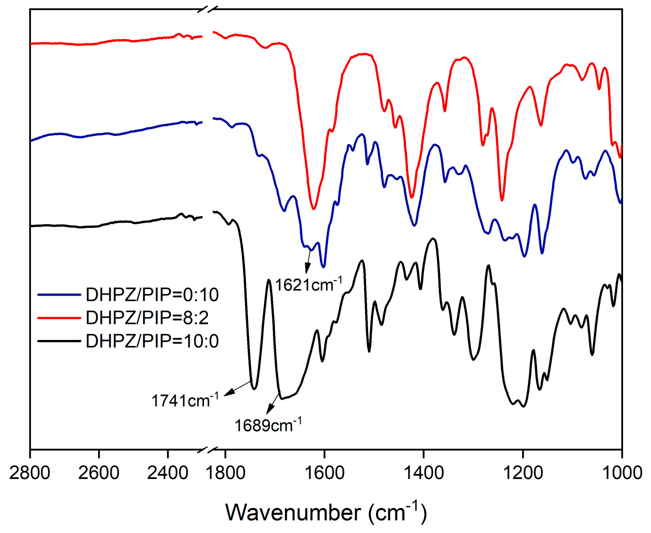

Fig. S1. FT-IR spectra of the fabricated films.

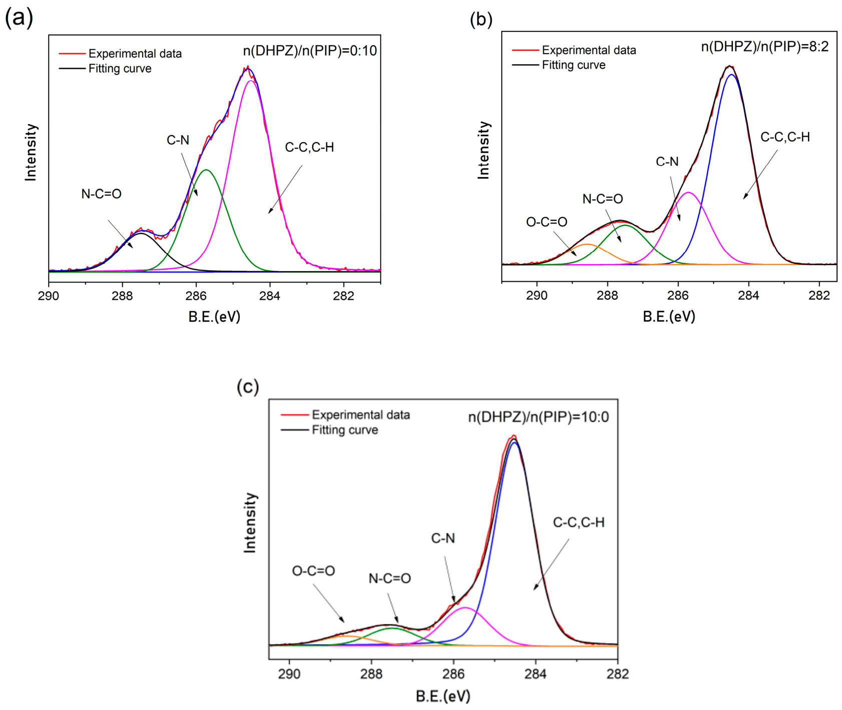

Fig. S2. C 1s XPS spectra of fabricated films (a) n(DHPZ)/n(PIP)=0:10; (b) n(DHPZ)/n(PIP)=8:2; (c) n(DHPZ)/n(PIP)=10:0.
